# Comparison of ^18^F-T807 and ^18^F-THK5117 PET in a Mouse Model of Tau Pathology

**DOI:** 10.3389/fnagi.2018.00174

**Published:** 2018-06-07

**Authors:** Matthias Brendel, Behrooz H. Yousefi, Tanja Blume, Michael Herz, Carola Focke, Maximilian Deussing, Finn Peters, Simon Lindner, Barbara von Ungern-Sternberg, Alexander Drzezga, Peter Bartenstein, Christian Haass, Nobuyuki Okamura, Jochen Herms, Igor Yakushev, Axel Rominger

**Affiliations:** ^1^Department of Nuclear Medicine, Ludwig-Maximilians-University of Munich, Munich, Germany; ^2^Department of Nuclear Medicine, Technical University of Munich, Munich, Germany; ^3^Neuroimaging Center, Technische Universität München, Munich, Germany; ^4^German Center for Neurodegenerative Diseases (DZNE), Munich, Germany; ^5^Department of Nuclear Medicine, University of Cologne, Cologne, Germany; ^6^Munich Cluster for Systems Neurology (SyNergy), Munich, Germany; ^7^Biomedical Center, Ludwig-Maximilians-University of Munich, Munich, Germany; ^8^Division of Pharmacology, Faculty of Medicine, Tohoku Medical and Pharmaceutical University, Sendai, Japan

**Keywords:** tau, small animal PET, transgenic mice, ^18^F-T807, ^18^F-THK5117

## Abstract

Positron-emission-tomography (PET) imaging of tau pathology has facilitated development of anti-tau therapies. While members of the arylquinoline and pyridoindole families have been the most frequently used tau radioligands so far, analyses of their comparative performance *in vivo* are scantly documented. Here, we conducted a head-to-head PET comparison of the arylquinoline ^18^FT807 and the pyridoindole ^18^FTHK5117 PET in a mouse model of tau pathology. PET recordings were obtained in groups of (*N* = 5–7) P301S and wild-type (WT) mice at 6 and 9 months of age. Volume-of-interest based analysis (standard-uptake-value ratio, SUVR) was used to calculate effect sizes (Cohen’s *d*) for each tracer and age. Statistical parametric mapping (SPM) was used to assess regional similarity (dice coefficient) of tracer binding alterations for the two tracers. Immunohistochemistry staining of neurofibrillary tangles was performed for validation *ex vivo*. Significantly elevated ^18^F-T807 binding in the brainstem of P301S mice was already evident at 6 months (+14%, *p* < 0.01, *d* = 1.64), and increased further at 9 months (+23%, *p* < 0.001, *d* = 2.70). ^18^F-THK5117 indicated weaker increases and effect sizes at 6 months (+5%, *p* < 0.05, *d* = 1.07) and 9 months (+10%, *p* < 0.001, *d* = 1.49). Regional similarity of binding of the two tracers was high (71%) at 9 months. ^18^F-T807 was more sensitive than ^18^F-THK5117 to tau pathology in this model, although both tracers present certain obstacles, which need to be considered in the design of longitudinal preclinical tau imaging studies.

## Introduction

Neurofibrillary tangles constitute one of the most characteristic neuropathological findings in Alzheimer’s disease (AD), which is the most frequent form of dementia, and likewise occur in certain non-AD forms of dementia known as tauopathies (Duyckaerts et al., 2009). Whereas AD is characterized by paired helical filaments containing equal amounts of 3R and 4R isoforms, non-AD tauopathies present with other tau ultrastructures and isoforms. For example, the pathology in progressive supranuclear palsy and corticobasal degeneration consists of 4R tau aggregating into straight filaments (Murray et al., 2014). The recent development of ^18^F-fluorinated radioligands for positron-emission-tomography (PET) studies of tau aggregates intensely accelerated research in the field of tau imaging in AD and non-AD tauopathies (Villemagne and Okamura, 2016). Members of the arylquinoline and pyridoindole structural families have been the most frequently used tau radioligands to date. Although tau PET has been hindered due to off-target binding of ligands to neuromelanin (Hansen et al., 2016) and MAO-B (Ng et al., 2017), both tracer classes can differentiate AD and non-AD tauopathy patients from healthy controls.

Small animal PET (μPET) has emerged as a useful tool for translational research involving *in vivo* imaging of β-amyloid and neuroinflammation markers in rodent models of neurodegenerative diseases (Zimmer et al., 2014; Brendel et al., 2015; Liu et al., 2015). However, there have been relatively few tau-PET studies in wild-type (WT) and transgenic mice (Maruyama et al., 2013; Okamura et al., 2013), due in part to concerns about the sensitivity of tau ligands for species-dependent isoforms and intracellular compartmentation of tau aggregates. We previously characterized the performance of μPET with the arylquinoline ^18^F-THK5117 in two transgenic mouse models of tau pathology (Brendel et al., 2016); this tracer showed elevated brain uptake in both types of model mice relative to WT mice, and brain uptake patterns correlated with autoradiography and immunohistochemistry findings of tau deposition. Nonetheless, the sensitivity of ^18^F-THK5117 was rather low, with a detection threshold of 10% tau/area, and quantitation was degraded by spillover from extracerebral structures compounded by rapid washout from brain.

In light of the previous findings, further research is needed to improve preclinical imaging of tau pathology in mouse models. Thus, the aim of the current μPET study was to compare representative members of the two major classes of tau radioligands with regard to their ability to track longitudinally the expression of tau pathology in the brain of P301S transgenic mice.

## Materials and Methods

### Radiochemistry

Radiosynthesis of the pyridoindole ^18^F-T807 was performed as previously described (Gao et al., 2015), with modifications for a fully automated preparation on Neptis perform module^1^ as specified in the Supplementary Methods. A radiochemical yield of >20% was achieved, with chemical and radiochemical (HPLC) purities of >98%. The ^18^F-T807 was directly used for μPET with a specific activity of >216 GBq/μmol at the end of synthesis.

Radiosynthesis of ^18^F-THK5117 was performed as previously described (Tago et al., 2014), with slight modifications as specified in the Supplementary Methods. The procedure yielded a radiochemical purity >98% and specific activity of 202 ± 56 GBq/μmol at the end of synthesis.

### Animals and Study Design

All experiments were carried out in compliance with the German national guidelines for animal protection (TierSchG, Germany) and approval of the local animal care committee (Regierung von Oberbayern), with supervision by a veterinarian. Animals were housed in a temperature- and humidity-controlled environment with a 12-h light–dark cycle, with free access to food (Ssniff, Soest, Germany) and water.

Tau-P301S (P301S) transgenic (TG) mice were investigated in this study together with age-matched WT controls. P301S mice express human P301S mutant 4R/0N tau (Thy1-hTau.P301S) in CBA.C57BL/6 background (Allen et al., 2002). This model is characterized by predominant tau hyperphosphorylation in the brainstem, where tau filaments appear mostly as half-twisted ribbons. Larger paired helical tau filaments reminiscent of those seen in human AD are observed less frequently. The behavioral phenotype manifests as learning deficits from 2 to 3 months of age, and onset of motor impairment at 4 months, leading to early death before 12 months of age. Eight P301S mice and eight age-matched C57Bl/6 littermates serving as controls were included in the study. Imaging with dual tracers was performed at baseline (at 6 months of age) and at follow-up (at 9 months of age). Maximum time span between paired measurements was 10 days.

### Tau PET

#### Data Acquisition and Analyses

**FIGURE 1:**
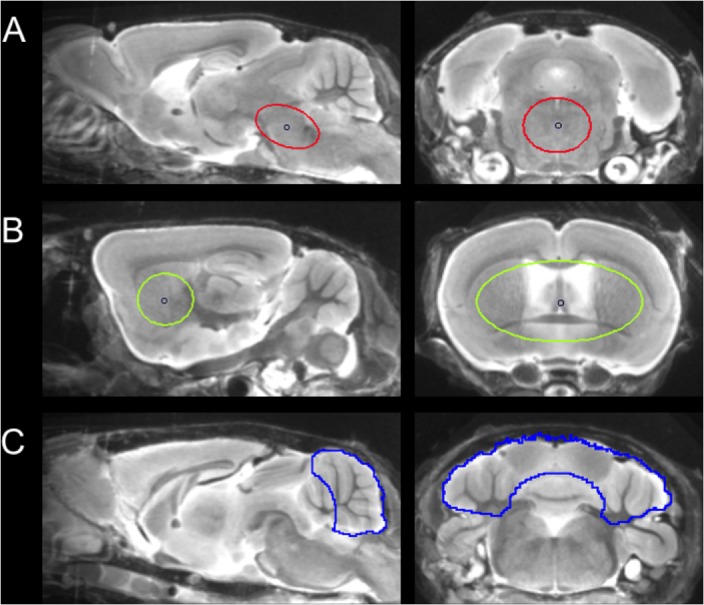
Definition of VOIs projected on the mouse brain MRI atlas (Dorr et al., 2007) in sagittal and coronal slices: **(A)** Oval-shaped brainstem VOI including central parts of the pons and the midbrain (middle row; 11 mm^3^; red line). **(B)** Spherical striatal reference VOI (top row; 38 mm^3^; green line). **(C)** Crescent-shaped cerebellar reference VOI (top row; 56 mm^3^; blue line).

For details of data acquisition and reconstruction see Supplementary Methods. In brief, summed 5–65 min dynamic emission frames for each tracer (^18^F-T807 and ^18^F-THK5117) were co-registered to an MRI mouse brain atlas (Dorr et al., 2007) after frame-wise correction for motion using the PMOD fusion tool (V3.5, PMOD Technologies Ltd.). The reader was blind to the type of mouse. A second experienced reader ensured accurate alignment of the fusion image and corrected if necessary. Volumes-of interest (VOI) were defined on the MRI mouse atlas (**Figure 1**).

Due to progressively increasing bone labeling with ^18^F-T807 scan duration, we modified our previously published brainstem VOI (Brendel et al., 2016) by reducing its width and increasing its length, so as to reduce signal spill-in from extra-cerebral structures near the ovoid VOI (11 mm^3^). Bone uptake was obtained from a bilateral VOI in the petrous bone (9 mm^3^) and uptake in the bilarteral Harderian glands was also measured (40 mm^3^). A whole brain VOI was applied to measure the radiotracer uptake in the entire brain (525 mm^3^). The cerebellum, with exclusion of voxels of the cerebellar peduncle (volume 56 mm^3^), which was the optimal reference VOI identified for ^18^F-THK5117, (Brendel et al., 2016), proved to be vulnerable to spill-in from the extensive bone uptake occurung in the ^18^F-T807 scans. Therefore we used the bilateral striata together with the septum (volume 38 mm^3^) as the reference tissue VOI, which was unaffected by cranial spill-in from ^18^F-T807, and is not reported to contain tau deposition in P301S mice (Allen et al., 2002). To enable direct comparisons of both tracers using the same reference region, we performed additional analyses with modified striatal and cerebellar reference regions, which are presented in the Supplementary Material. Time-activity-curves of brainstem, striatum, cerebellum, Harderian glands, and petrous bone were extracted from the dynamic emission sequences. Brainstem and reference VOIs were employed for calculation of the mean brainstem-to-striatum ratio for ^18^F-T807 (40–60 min, SUVR_BST/STR_) and the brainstem-to-cerebellum ^18^F-THK5117 (30–60 min, SUVR_BST/CBL_), where SUVR is the standardized uptake value ratio. A reader-independent coregistration was established after validation of the optimal time window by stability analysis of ratio TACs in the emission sequence for each tracer. Further details are provided in Supplementary Methods.

### Statistical Parametric Mapping (SPM)

For whole-brain voxel-wise comparisons of the two tau tracers, SPM was performed using SPM5 routines (Wellcome Department of Cognitive Neurology, London, United Kingdom) implemented in MATLAB (version 7.1) (Rominger et al., 2013). SUVR images were scaled by the tracer-specific reference region before statistical analysis.

### Immunohistochemistry

After completion of the final μPET scans, mice were killed by cervical dislocation, and the brains rapidly removed. After fixation by immersion overnight in 4% paraformaldehyde at 4°C, brains were post-fixed in phosphate buffered saline. Two representative 50 μm thick slices per animal were cut in the sagittal plane using a vibratome (VT 1000 S, Leica, Wetzlar, Germany). Free-floating sections were permeabilized with 2% Triton X-100 overnight and blocked with I-Block^TM^ Protein-Based Blocking Reagent (Thermo Fischer Scientific). We obtained immunohistochemical labeling of microtubule-associated protein tau (MAPT) using PA5-27287 primary antibody (Invitrogen, dilution 1:400) – recognizing amino acids 1 – 286 of human Tau and the A-21244 secondary antibody (Invitrogen, 1:500). The unbound dye was removed by three washing steps with PBS, and the slices were then mounted on microscope slides with fluorescent mounting medium (Dako, Germany). Images were acquired with a LSM 780 confocal microscope (Zeiss) equipped with a 40×/1.4 oil immersion objective. The excitation wavelength was 633 nm and emission was detected from 650 to 758 nm. For each brain slice 3-dimensional 16 bit data stacks of 10240 pixels × 9216 pixels × 67 pixels were acquired from the brainstem (area matching the VOI of PET) at a lateral resolution of 0.35 μm/pixel and an axial resolution of 1.0 μm/pixel. Whole brain overview images were obtained by two-dimensional acquisition.

To quantify tau volume load, we employed custom-written Matlab software. Local background subtraction was used to diminish intensity variations among different stacks. Subsequently, MAPT was identified by applying the 80th percentile as minimal intensity threshold. Noise was excluded by applying a connected component analysis excluding patches of contiguous voxels smaller than 1 μm. Within this tau 3D mask contiguous patches with a minimum diameter of 3 μm in all spatial directions were considered as tau-positive somata. These analyses were performed by an operator who was blind to the μPET results.

### Statistics

Group comparisons of VOI-based PET results between TG and WT mice (baseline and follow-up) were assessed by an unpaired Student’s *t*-test using IBM SPSS Statistics (version 23.0; SPSS, Chicago, IL, United States). Longitudinal data were compared between baseline and follow-up by a paired Student’s *t*-test. A threshold of *p* < 0.05 was considered to be significant for rejection of the null hypothesis.

## Results

### Overview

One P301S and one WT mouse died before the first PET scan. Two P301S mice had to be euthanized after the follow-up ^18^F-T807 scan due to poor health and did not receive the second ^18^F-THK5117 scan. Resulting final animal numbers and PET SUVR results by group are presented in **Table 1**.

**Table 1:** Demographics and result overview.

PET session	Age (months)	SUVR P301S	SUVR WT	Effect size (*d*)
BL ^18^F-T807	6	1.11 ± 0.07^∗∗^	0.97 ± 0.09	1.64
		(*N* = 7)	(*N* = 7)	
FU ^18^F-T807	9	1.18 ± 0.04^∗∗∗^	0.95 ± 0.07	2.70
		(*N* = 7)	(*N* = 7)	
BL ^18^F-THK5117	6	1.11 ± 0.04^∗^	1.06 ± 0.05	1.07
		(*N* = 7)	(*N* = 7)	
FU ^18^F-THK5117	9	1.16 ± 0.05^∗∗∗^	1.06 ± 0.09	1.49
		(*N* = 5)	(*N* = 7)	


*BL, baseline; FU, follow-up. P301S versus WT: ^∗^*p* < 0.05; ^∗∗^*p* < 0.01; ^∗∗∗^*p* < 0.001; *d*, Cohen’s *d*.*

### Comparison of Tracer Characteristics

**FIGURE 2:**
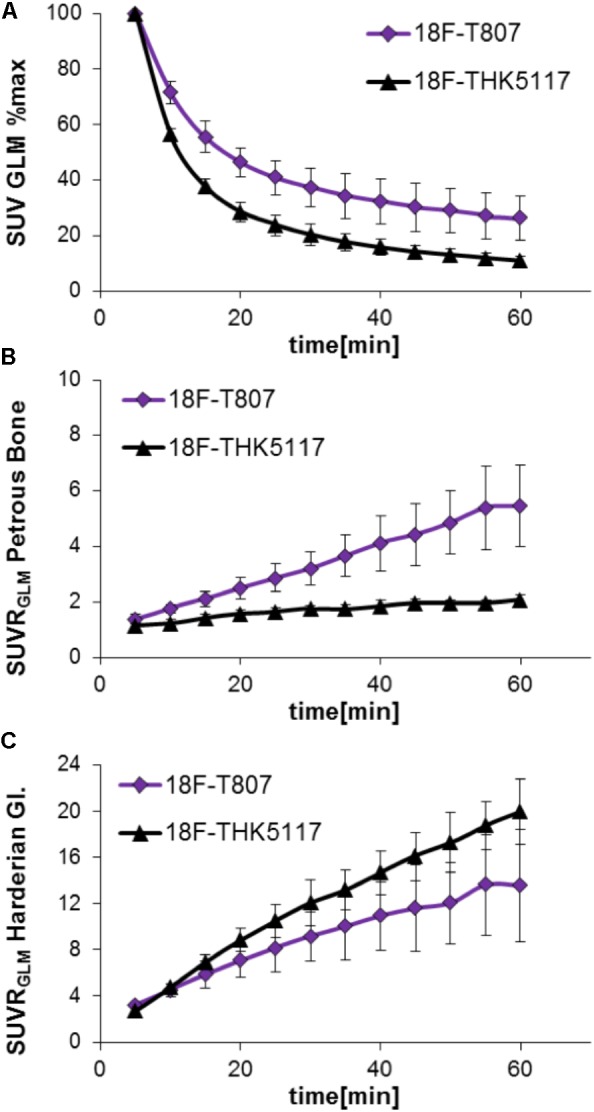
Comparison of ^18^F-T807 and ^18^F-THK5117 wash-out from brain **(A)** and uptake in adjacent extracerebral structures (**B**: Petrous bone; **C**: Harderian glands).

Head-to-head comparison of the TACs indicated a faster tracer washout of ^18^F-THK5117 from whole brain when compared to ^18^F-T807. Relative to the mean peak activity, 57% remained at 10 min and 11% remained at 60 min after ^18^F-THK5117 injection, while 72% remained at 10 min and 26% at 60 min after ^18^F-T807 injection (both contrasts *p* < 0.001; **Figure 2A**). Radioactivity uptake in the petrous bone relative to the whole brain uptake was 42% higher for ^18^F-T807 than ^18^F-THK5117 (*p* < 0.001) at 10 min post injection (p.i.), and +164% higher at 60 min p.i. (p < 0.001) (**Figure 2B**). ^18^F-THK5117 uptake in the Harderian glands relative to the whole brain significantly exceeded that for ^18^F-T807 after 15 min p.i. (+15%, *p* < 0.05), with a widening gap between the two tracers until 60 min p.i. (+32%, *p* < 0.001) (**Figure 2C**). A previously reported frontal cortex hotspot (Brendel et al., 2016) was evident in 31% of ^18^F-THK5117 SUVR images, but was absent in all ^18^F-T807 scans.

### Dynamic Tau Imaging

**FIGURE 3:**
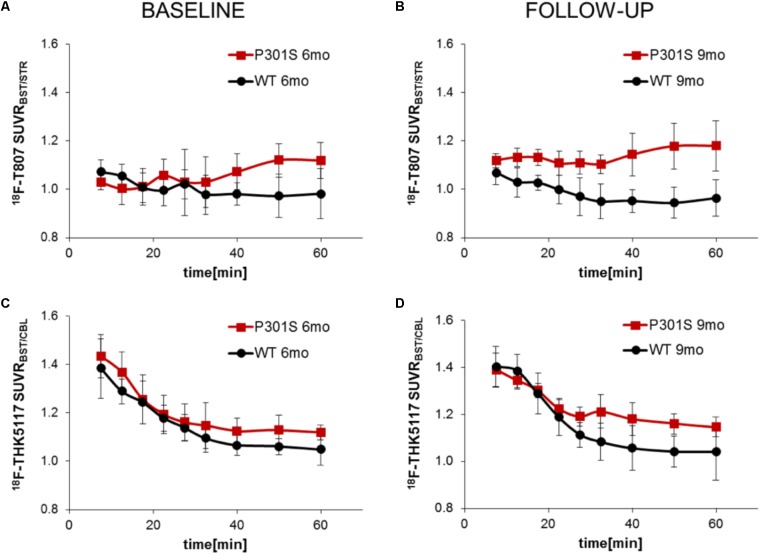
Curves of ratios over time derived from dynamic PET imaging of ^18^F-T807 **(A,B)** and ^18^F-THK5117 **(C,D)**. Red lines/squares show transgenic P301S mice and black lines/circles indicate WT mice. Each point is the mean of 5–7 measurements, with error bars indicating SD.

TAC ratios (SUVR_BST/STR_) for ^18^F-T807 stabilized after 30 min p.i. in baseline and follow-up scans, with the 40–60 min p.i. interval period giving the best discrimination between P301S and WT mice at 6 and 9 months of age (**Figures 3A,B**). TAC ratios (SUVR_BST/CBL_) of ^18^F-THK5117 also stabilized after 30 min p.i. for baseline and follow-up time scans, but the 30–60 min period p.i. gave the best discrimination between P301S and WT mice (**Figures 3C,D**).

### VOI and SPM Analyses of Static SUVR Images

**FIGURE 4:**
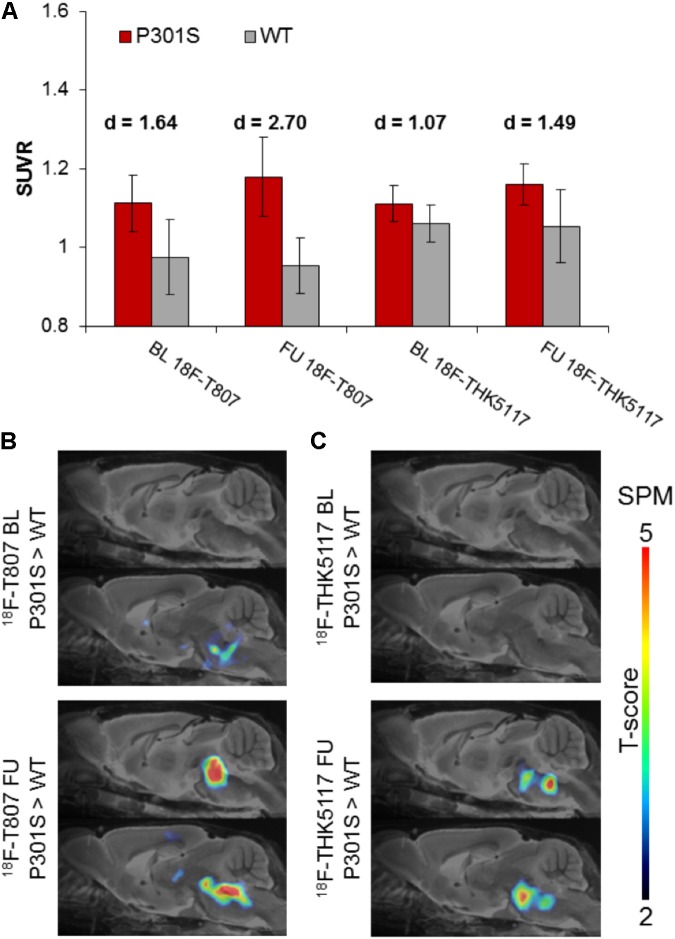
**(A)** Bar graphs show group mean SUVRs of P301S (red) and WT (gray) mice for baseline and follow-up PET measurements of ^18^F-T807 and ^18^F-THK5117. Error bars indicate SD, effect sizes are given by Cohen’s *d*. Sagittal slices (median and 0.6 mm paramedian) show voxel-wise statistical parametric mapping (SPM) between transgenic P301S and WT mice at baseline (BL) and follow-up (FU) for ^18^F-T807 **(B)** and ^18^F-THK5117 **(C)**. SPM-maps are depicted upon a MRI mouse atlas and extracerebral voxels are masked. The *t*-score threshold of 2 complies a significance threshold of 0.01 uncorrected.

^18^F-T807 indicated significantly higher uptake in the brainstem VOI of P301S mice when compared to WT mice at baseline (+14%, *p* < 0.01) and follow-up PET scanning (+23%, *p* < 0.001). Brainstem uptake of ^18^F-THK5117 was slightly elevated at baseline (+5%, *p* < 0.05) in the same contrast, and increased further at follow-up (+10%, *p* < 0.05). Effect sizes were distinctly higher for ^18^F-T807 brainstem uptake when compared to ^18^F-THK5117 at both scanning ages (**Figure 4A**). Utilization of the same reference regions resulted in a loss of the significant results for ^18^F-T807 when the modified cerebellum was used, and for ^18^F-THK5117 when the modified striatum was used (see Supplementary Results). The modified cerebellar reference region appeared to be generally less affected by bone uptake for ^18^F-T807, but the substantial bone uptake in some animals distinctly increased the variance and made group results unreliable. Even a modified striatal VOI degraded the otherwise significant results for ^18^F-THK5117, most likely due to hot spot noted above. SPM congruently showed higher *T*-scores for baseline and follow-up contrasts of ^18^F-T807 in comparison to ^18^F-THK5117 (**Figures 4B,C**). A comparison of T-score maps of both tau ligands (P301S vs. WT; 9 months) indicated a high dice similarity of 71%.

### Longitudinal Imaging of Tau Pathology in P301S Mice

**FIGURE 5:**
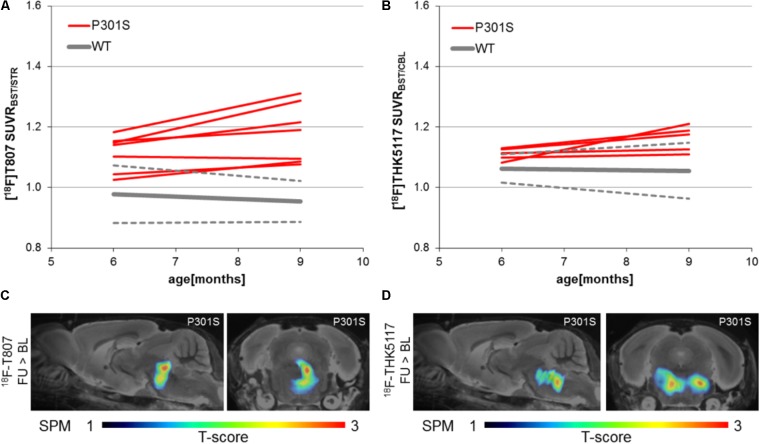
Longitudinal findings of SUVR for **(A)**^18^F-T807 and **(B)** ^18^F-THK5117 in individual WT and TG mice. Red lines represent SUVR of individual P301S mice at baseline and follow-up scanning. WT mice are presented in gray by group mean (solid) ± SD (dotted). Sagittal (0.6 mm paramedian) and coronal (pons/midbrain junction) slices show voxel-wise results of longitudinal statistical parametric mapping (SPM) showing regions with significantly increased tau ligand binding in transgenic P301S mice (**C**: ^18^F-T807; **D**: ^18^F-THK5117). SPM-maps are projected upon a MRI mouse atlas and extracerebral voxels are masked. The *t*-score threshold of 1 complies a significance threshold of 0.05 uncorrected.

Longitudinal increases in SUVR between baseline and follow-up scanning in P301S mice were 5.9 ± 4.5% for ^18^F-T807 (*p* < 0.05) and 4.7 ± 4.4% for ^18^F-THK5117 [*p* = not significant (n.s.)]. SPM indicated comparable longitudinal changes in the brainstem of P301S mice by both tracers. (**Figures 5C,D**). WT mice did not show any longitudinal changes in SUVR for either tracer.

### Immunohistochemical Validation

**FIGURE 6:**
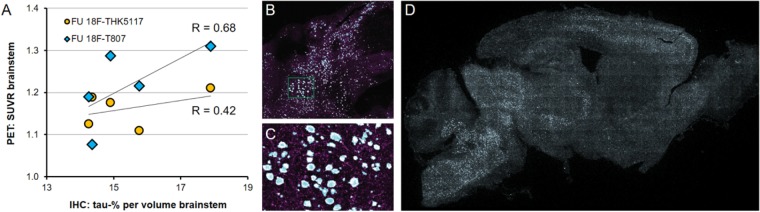
**(A)** PET-Immunohistochemistry correlation of ^18^F-T807 (blue diamonds) and ^18^F-THK5117 (yellow circles) SUVR with %tau/area to PA5-27287 antibody staining. **(B)** 3-dimensional brainstem acquisition of PA5-27287 staining and **(C)** zoomed image indicating automatically detected tau positive cell somata. **(D)** 2-dimensional whole brain overview shows tau-positive cells predominantly in the brainstem, and lower amounts in the neocortex. Data derive from *N* = 5 P301S mice.

P301S mice revealed tau-positive staining predominantly in the brainstem, and penetrating into the cerebellar peduncles, with lesser amounts in the forebrain, and complete absence in the cerebellar hemispheres, as previously demonstrated (Brendel et al., 2016). Similarly, the striatum did not indicate any tau deposition to immunohistochemistry. The tau burden was 15.4 ± 1.5% per volume in the brainstem by the new 3-dimensional approach. %-tau positively correlated with SUVR of ^18^F-T807 (*R* = 0.68, *p* = 0.21) and SUVR of ^18^F-THK5117 (*R* = 0.42, *p* = 0.48), although these associations did not reach significance (**Figure 6**).

## Discussion

This is to our knowledge the first longitudinal small animal PET study of tau distribution in a transgenic mouse model of tau pathology, comprising an *in vivo* comparison of representatives of the two currently most frequently used tau PET tracers. Quantitation of the pyridoindole ligand ^18^F-T807 and the arylquinoline ligand ^18^F-THK5117 present specific obstacles for preclinical PET imaging, arising from the substantial uptake in bone and Harderian glands, resulting in signal spill-in, compounded by especially fast washout of brain signal from ^18^F-THK5117. In addition, we again observed the inexplicable accumulation of ^18^F-THK5117 at the frontal pole of some mice, irrespective of the genotype. Despite these issues, the current data provide compelling evidence that longitudinal preclinical PET imaging of tau pathology in P301S mice is feasible with both radioligands. We found moderately superior sensitivity of ^18^F-T807 for the contrast of P301S versus age-matched WT mice, compared to ^18^F-THK5117. Voxel-wise analysis of the age-dependent increases in SUVR in P301S mice indicated high regional similarity for the two ligands, suggesting that they are both sensitive to tau accumulation in the brainstem.

### Head to Head Comparison of ^18^F-T807 and ^18^F-THK5117

A strength of this study lies in the head-to-head dual tau tracer assessment in the same animals examined within a span of only a few days. By this approach, we were able to directly compare ^18^F-T807 and ^18^F-THK5117 labeling of tau pathology in the brainstem of P301S mice at two different ages. Higher effect sizes were observed with ^18^F-T807 for baseline and follow-up comparisons of P301S versus WT groups. Thus, considering results of the baseline scans, the detection threshold of tau burden (%/area) in the brainstem of P301S mice is evidently lower for ^18^F-T807 than for ^18^F-THK5117. This is in accord with results of a recent study showing higher sensitivity for ^18^F-AV-1451 in a head-to-head comparison ^18^F-THK5351 by PET imaging of AD patients *in vivo* (Jang et al., 2017). As histological tau burden is already present at the age of 6 months in these mice (Brendel et al., 2016), it seems that both ligands are practically insensitive to early tau deposition in the rodent brain. A limitation of our comparison lies in the impossibility of using exactly the same reference region for both tracers. While reasons for this difficulty are discussed below, we are convinced that using the optimal reference tissue for either tracer is a valid approach. Both reference tissues were lacking in tau pathology to immunohistochemical analyses, which substantiates their suitability, despite the lack of a single region optimal for both tracers. In general, the signal of both radioligands was only moderately increased (versus WT) at the follow-up PET scan at 9 months of age, despite the very heavy tau burden in the brainstem of P301S mice at this age (14–18%). The lower histological tau concentrations in the frontal cortex were not detected by either PET ligand irrespective of the TG mouse age. In this regard, present results are consistent with our previous ^18^F-THK5117 investigation in the same TG mouse model (Brendel et al., 2016), although we note that percentages of the newly developed 3-dimensional approach fell distinctly below the earlier 2-dimensional immunohistological assessment. This is surely related to overlay effects arrising from a 2-dimensional acquisition, were several cell layers are summed in the calculation (illustrated in **Figures 6B–D**).

There is still some controversy about the specificity of pyridoindole and arylquinoline ligands for non-AD tau, with conflicting findings reported *in vivo* and *in vitro* (Chiotis et al., 2016; Ishiki et al., 2016; Smith et al., 2016). Aged P301S mice contain 4R tau, mainly occurring in a half-twisted ribbon conformation typical of human frontotemporal dementia, but they also express paired-helical filaments similar to those occurring in AD (Allen et al., 2002). Although both radioligand classes showed binding to 4R non-AD tau in brain of patients with corticobasal degeneration or progressive supranuclear palsy *in vivo* (Josephs et al., 2016; Hammes et al., 2017; Smith et al., 2017), and likewise to *post mortem* examination (Josephs et al., 2016; Kikuchi et al., 2016), other studies did not find significant binding in 4R-tauopathies, e.g., using ^18^F-T807 (Coakeley et al., 2016; Marquie et al., 2017). The ultrastructure of tau as expressed in P301S mice might not be perfectly suited for high affinity binding by ^18^F-T807 and ^18^F-THK5117. This issue will only be circumvented if sensitive and specific ligands for the 4R form of tau are developed. For the present, translational comparisions between tracer binding to tau in mice and humans should be interpreted with caution as tau ultrastructures in mouse models only partially match the human pathology.

We have also evaluated the arylquinoline ^18^F-THK5351 in mouse models of tau pathology and WT mice. However, ^18^F-THK5351 proved to have such rapid kinetics in mouse brain that specific tau signal in brain was overwhelmed by spill-in from extra-cerebral regions (see Supplementary Results). Thus, we elected to use the previsously evaluated arylquinoline^18^F-THK5117 for the current study.

### Challenges of Preclinical Tau PET

Bone uptake is more pronounced for ^18^F-T807 than to ^18^F-THK5117 (**Figure 2**), but extracerebral binding must be taken into consideration when planning the target and reference region of preclinical tau PET studies with either of these ligands. In general, bone labeling is indicative of hepatic defluorination of the tracer, which is often more pronounced in rodents than in humans or non-human primates. Present results suggest that tracking of mouse cortical tau pathology may be favored with ^18^F-THK5117, due to the less contribution of signal spill-in than from ^18^F-T807. On the other hand, imaging of tau in the frontal pole of the cortex is problematic for ^18^F-THK5117 due to spill-over from the Harderian glands, and the still inexplicable hot-spot signal in about one third of mice with C57Bl/6 background, irrespective of their transgenic status. Mice of other backgrounds do not seem to be affected by the latter phenomenon (Brendel et al., 2016). The current brainstem target region (in the modified version) did not indicate clear advantages or disadvantages for one or the other of the ligands, although we chose in the present study to alter its shape so as to better avoid spill-over from the petrosal bone, which is pronounced in the case of ^18^F-T807 μPET.

Another difficulty presented by tau PET with the arylquinoline ^18^F-THK5351 and also the pyridoindole ^18^F-T807 is off-target binding to monoamine oxidases (MAO) A or B (Vermeiren et al., 2015; Ng et al., 2017). A recent study has also indicated off-target binding of ^18^F-THK5117 to MAO-B in brain (Lemoine et al., 2017), which might have been predicted from structural motifs. However, the contribution of MAO to the ^18^F-THK5117 binding signal in mouse brain remains to be established. Additional *in vivo* blocking experiments by MAO inhibitors were not possible in the present cohort due to the high rate of drop-outs expected for aged TG exposed to additional scanning sessions. Thus, we cannot presently exclude a contribution of MAO binding to the present findings with ^18^F-T807 and ^18^F-THK5117 PET, although we note that the immunohistochemical findings support our attribution of the PET results mainly to tau deposition. Most relevantly, MAO can be elevated as part of inflammatory responses in the course of neurodegenerative diseases (Carter et al., 2012). If parts of the tracer signal in our tau model indeed belongs to MAO binding, it will be necessary to separate the binding components by pharmacological blocking studies, or in double TG mice with knockout of MAO A or B. Due to the configuration of our animal holder, we are unable to obtain blood samples during the recording. This introduces some variance due to individual differences in tracer metabolism, although our use of SUVR values should largely compensate for such factors.

### Longitudinal Imaging

Present data give a first demonstration of the value of longitudinal μPET imaging in tau transgenic models. The phenotype of mouse models of neurodegenerative diseases is well-known to show considerable heterogeneity, as seen in the present study from the different baseline SUVR results of P301S mice for both tau tracers in **Figure 5**. By recording the kinetics of tau deposition in individual animals it should in future studies be feasible to minimize possible biasing effects of differing pathological trajectories depending on baseline differences. This scenario is reminiscent of our earlier preclinical findings with amyloid-PET, whereby individual responses to an anti-amyloid treatment proved to be dependent on the amyloidosis at baseline (Brendel et al., 2015). Thus, PET imaging of P301S mice can be translationally used to monitor anti-tau drugs during preclinical evaluation and this data will potentially lead to a better understanding of tau-PET monitoring in humans. As a limitation we note that longitudinal increases of MAO could as well lead to a elevated PET signal due to off-target binding, however, recent data in AD suggests different time courses for astrocytosis and tau protein deposition (Scholl et al., 2015).

## Conclusion

Longitudinal μPET imaging of tau pathology is feasible in a preclinical setting using P301S mice. Analysis of brainstem uptake showed ^18^F-T807 to be moderately superior to ^18^F-THK5117 regarding sensitivity for preclinical tau imaging. The two tracers, which represent distinct structural classes, both suffer from particular shortcomings in the present mouse model, which must be considered in the design of longitudinal studies of tau deposition.

## Author Contributions

MB and BY organized and executed the research project, executed the statistical analysis, and wrote the first draft of manuscript. TB, MH, CF, MD, FP, SL, and BvU-S executed the research project and reviewed and critiqued the manuscript. AD, CH, and JH reviewed and critiqued the statistical analysis. AD, CH, JH, PB, and NO reviewed and critiqued the manuscript. IY and AR conceived and organized the research project, designed, reviewed, and critiqued the statistical analysis, and reviewed and critiqued the manuscript.

## Conflict of Interest Statement

The authors declare that the research was conducted in the absence of any commercial or financial relationships that could be construed as a potential conflict of interest.

## Footnotes

1 http://www.neptis-vsa.com/
