# Supplementary material for: Comparison of 18F-T807 and 18F-THK5117 PET in a Mouse Model of Tau Pathology
Source: Front Aging Neurosci. 2018 Jun 7;10:174. doi: 10.3389/fnagi.2018.00174 (PMC5999706; doi:10.3389/fnagi.2018.00174)
Supplement: Supplementary file 1 [file Data_Sheet_1.DOCX]

**Supplementary Material**

**Supplementary Methods**

**Radiosynthesis ^18^F-T807**

The nitro precursor, 7- (6-nitropyridin-3-yl) -5H-pyrido [4,3-b] indole, was obtained from API-Consult (NPPI-95-0100, L-9991 Weiswampach, Luxembourg) and characterized by HPLC-MS (≥ 99% purity). The chemicals and solvents for the synthesis were purchased from the suppliers (VWR and Sigma-Aldrich) or the pharmacy of the clinic (water for injection purposes, saline solution) and used without any further purification.

The ^18^F-T807 synthesis is carried out by a fully automated radiosynthesis of the nitro-precursor 7-(6-nitropyridin-3-yl) -5H-pyrido [4,3-*b*] indole with ^18^F-fluoride in a Neptis module (see Suppl. Fig.1). After the radiosynthesis, the product is purified by semi-preparative HPLC (Zorbax, Agilent) using 50% ethanol and 10 mM ammonium formate. The product fraction is then mixed with water in the ratio 1: 6 and the product is extracted by means of solid phase extraction (SampliQ OPT 30 mg (Agilent)). Then, the cartridge is rinsed with 10 ml of water for injection purposes and then the product is eluted from the cartridge with 1 ml of ethanol (96%). The eluate is diluted with isotonic saline solution and introduced to the animals in µpet.

The quality control of ^18^F-T807 includes analysis of external appearance (clear solution, particle free), pH value (6.0 - 8.0), radioactive concentration (100-300 MBq / ml), identity (retention time HPLC, corresponds to the retention time of the reference compound), content ^19^F-T807 (HPLC) ≤ 1.0 μg / ml, radiochemical purity of ^18^F-T807 (radio-HPLC) ≥ 98%; radiochemical purity ^18^F-T807 (radio-TLC) ≥ 95%, bacterial endotoxins <17.5 EU / ml, radio nuclear identity (γ-spectroscopy): a peak at 0.511 MeV ± 0.002 MeV (and sum peak at 1.022 MeV) radionuclear purity (γ-spectroscopy) ^18^F-fluorine ≥ 99.9%, as well as sterility (germ-free, no growth).


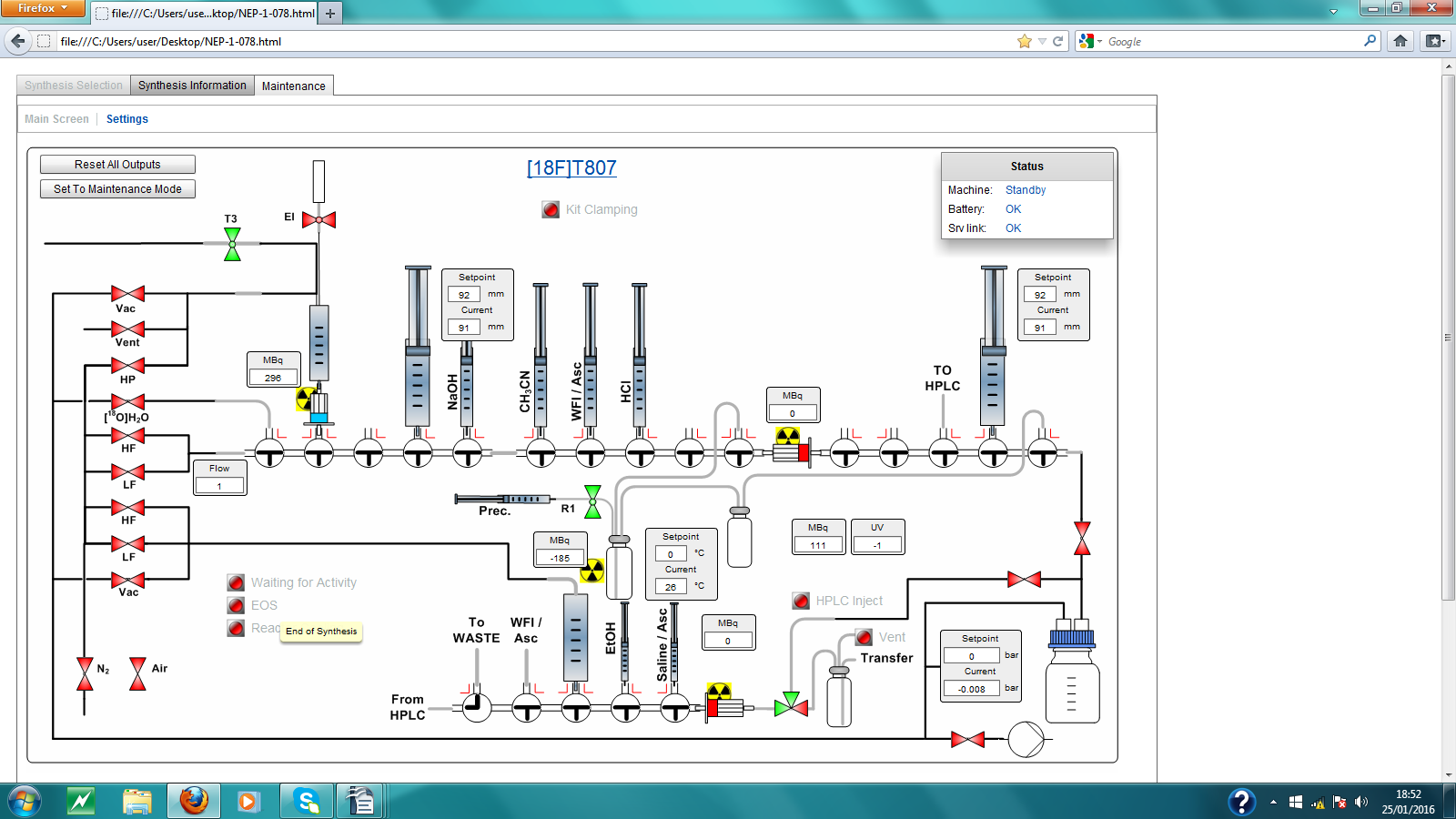


**Suppl. Fig.1** The fully automated radiosynthesis of ^18^F-T807 chart using the Neptis module

**Radiosynthesis ^18^F-THK5117**

Automated production of ^18^F-THK5117 was performed on a Raytest® SynChrom R&D single reactor synthesizer. Solvent containers (SC) were loaded with reagents, and cartridges were assembled on the synthesizer. The manufacturing process was performed automatically using the Raytest^®^ control software. No-carrier-added ^18^F-fluoride was produced via ^18^O(p, n)^18^F reaction by proton irradiation of ^18^O-enriched water and directly delivered to an ion exchange cartridge (Chromabond PS-HCO_3_-, Macherey Nagel, Trap 1). The trapped ^18^F-fluoride was eluted into the reactor using a mixture of Kryptofix®222 (12.5 mg), potassium carbonate (12.5 µL, 1 M), water (187.5 µL) and acetonitrile (800 µL) from SC 2. The solution was evaporated to dryness by azeotropic distillation, and the drying process was repeated after addition of acetonitrile (0.8 mL) from SC 3. The precursor (2 mg) in DMSO (0.7 mL) was transferred from SC 1 to the reactor, and the mixture was heated at 110°C for 10 min. HCl (0.2 mL, 2 M) from SC3 was then added, and the mixture stirred for three min. at 110°C. After quenching with AcOK (0.1 mL, 4 M) in H_2_O (5 mL) from SC 4, the mixture was transferred to a SepPak tC18 Plus Short cartridge (Waters, Trap 2), which was then washed with H_2_O (5 mL, SC wash). Radioactive products were eluted with EtOH/H_2_O 1:1 (4 mL, SC Elute) and purified via semi-preparative HPLC (Inertsil ODS-4 C18 column, 250 x 10 mm, 5 µm; isocratic elution with 55% NaH_2_PO_4_ (20 mM) / 45% acetonitrile; flow: 5 ml/min; UV-detection: 360 nm). The HPLC purified product peak was collected in SC 11, diluted with H_2_O (20 mL) and ascorbic acid (0.5 mL, 25%) from SC 8 and passed through a tC18 SepPak Plus Short cartridge (Waters, Trap 3). The radiolabelled product was eluted with anhydrous ethanol (1 mL) from SC 7 into the product vial, diluted with 0.9% saline (9 ml) from SC 9 and filtered through a sterile filter (Acrodisc®, 0.2 µm, PALL). The RCY was 16±2% (n=8) and RCP 99% with 75 min synthesis time. Purity was confirmed via analytical HPLC (Inertsil ODS-4 C18 column, 150 x 4.6 mm, 5 µm; isocratic elution with 50% NaH_2_PO_4_ (20 mM) / 50% acetonitrile; flow: 1.5 ml/min; UV-detection: 360 nm).

**Tau-PET Data Acquisition and Analyses**

Mice were anesthetized with isoflurane (1.5%, delivered at 3.5 l/min) and placed in the aperture of the Siemens Inveon DPET ([Visser *et al.*, 2009](#_ENREF_27)) as described previously ([Rominger *et al.*, 2010](#_ENREF_21)). P301S mice and age-matched C57Bl/6 controls were scanned in a nearly dynamic setting: 5 min upon injection to a tail vein of 15.9 ± 2.4 MBq ^18^F-THK5117 or 15.7 ± 2.2 MBq ^18^F-T807 in 150 µl saline, an emission recording lasting 60 minutes was initiated, followed by a 15 min transmission scan using a rotating ^57^Co point source. Dynamic emission acquisitions consisted of 12 frames (12 x 5 min). Reconstruction was performed with 4 OSEM3D and 32 MAP3D iterations, and a zoom factor of 1.0, with scatter-, attenuation-, and decay-correction, resulting in a final voxel dimension of 0.78 x 0.78 x 0.80 mm. Following recovery from anaesthesia, mice were returned to their home cages, or were killed by cervical dislocation while still deeply anesthetized, prior to rapid brain removal.

**Tau-PET Reader Independent Coregistration**

To this end, SUV images were generated in this frame for all mice after the final manual MRI-atlas coregistration (TX_rigid_). Attenuation and decay-corrected images of all TG and WT mice were averaged to generate a 30-60 min standard template for each of the three tracers. Non-linear brain normalization was performed with the PMOD fusion tool for all single frame SUV images coregistered to the MRI atlas to obtain transformation (TX_BrainNorm_) for each mouse brain to the template. The manual (TX_rigid_) and automatic (TX_BrainNorm_) transformations were concatenated and applied to the native space µPET data to guarantee a minimum of interpolation. As the µPET templates had been initially aligned to the MRI mouse brain atlas, all final fused µPET images had the voxel dimensions of the MRI mouse brain atlas, i.e. 0.064 x 0.064 x 0.064 mm.

**Supplementary Results**

**Modified reference regions**


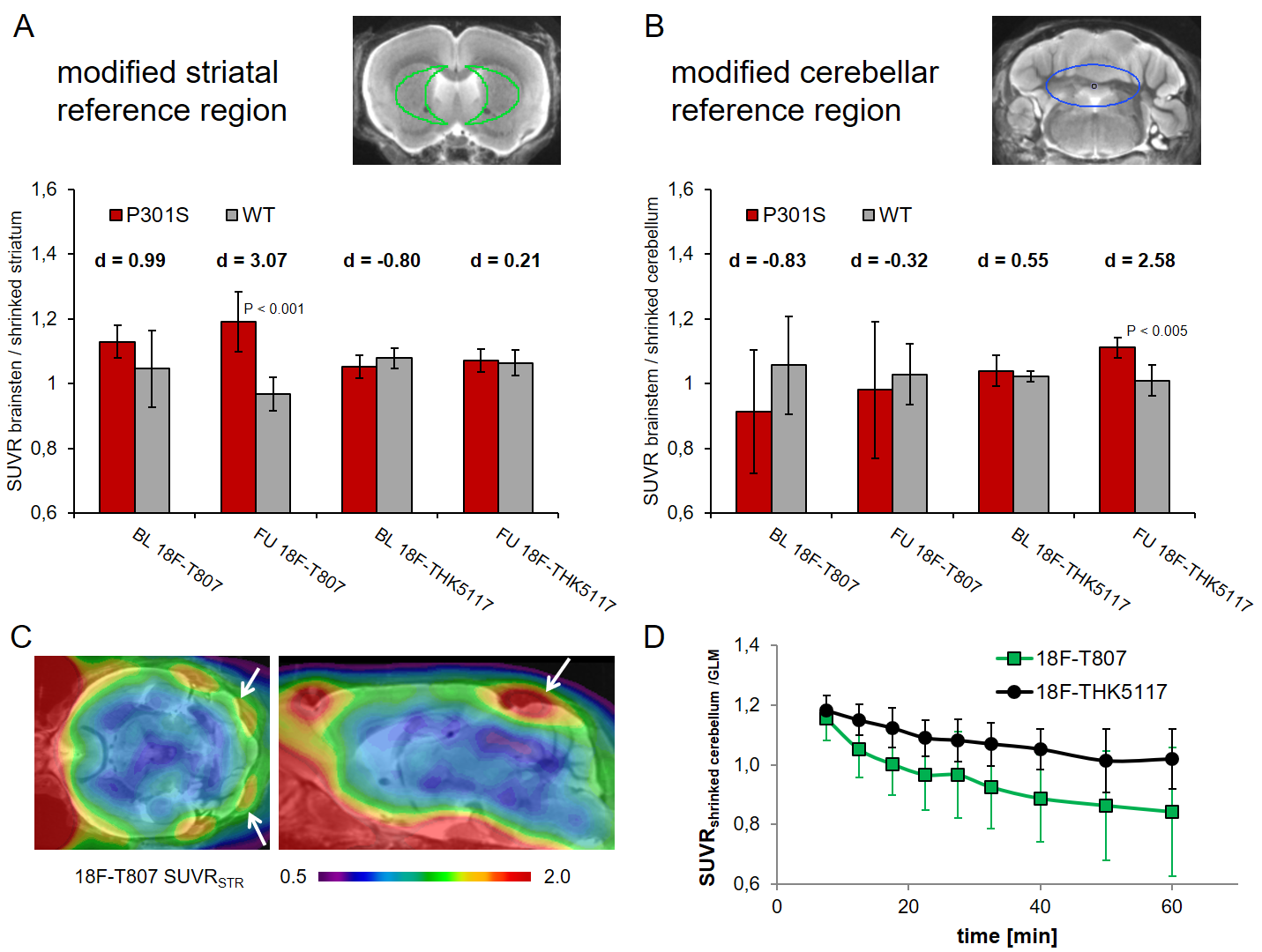


**Suppl. Fig.2** Analyses with modified reference regions applied to both tau tracers. (**A**) Parts of the striatal VOI that were visually affected by the frontal pole hot spot in ^18^F-THK5117 PET were excluded. Results still indicated significant differences for the follow-up comparison of P301S mice versus WT by ^18^F-T807 with the modified striatum reference VOI. However, the significance for the baseline comparison in the same contrast was lost, probably due to increasing variance by the smaller striatal reference region. Although we did not observe a large increase of variance in ^18^F-THK5117 results, the SUVR calculations by the modified striatum as a reference region degraded the significance of baseline or follow-up analyses of P301S mice versus WT, in contrast to the more robust findings with the cerebellar reference region (compare to Figure 4).

(**B**) We defined a cerebellar VOI using central parts of the cerebellum to avoid voxels that are affected by bone uptake in ^18^F-T807 PET. Variance of baseline and follow-up comparisons of P301S versus WT mice by ^18^F-T807 with this modified cerebellar reference region increased dramatically due to the variable extent of bone labelling in individual mice. Results for ^18^F-THK5117 indicated a lower effect size at baseline, which is consistent with known early tau-deposition in the cerebellar peduncle that probably increased the magnitude of the denominator. Interestingly, we observed an increasing effect size at follow-up for ^18^F-THK5117. This was most likely attributable to the lower variance of cerebellar voxels located further away from the brain’s edge (compare to Figure 4). Histological findings support this conjecture, as tau deposits in the brainstem came to exceed those of the cerebellar peduncle at late stages.

(**C**) Axial and sagittal slices of ^18^F-T807 PET (average of all scans) projected upon an MRI template show heavy bone labelling.

(**D**) TAC (average of all scans) for the modified cerebellar reference region using global mean normalization. Mean values of ^18^F-T807 do not indicate an increase with scan time in comparison with ^18^F-THK5117, which can be used as a surrogate for low bone labelling (compare to Figure 2B). However, variance of ^18^F-T807 increased strongly with greater scan time as bone labelling differed between individual animals and led to distorted and variable SUVR results (**B**).

**Comparison between ^18^F-THK5117 and ^18^F-THK5351**


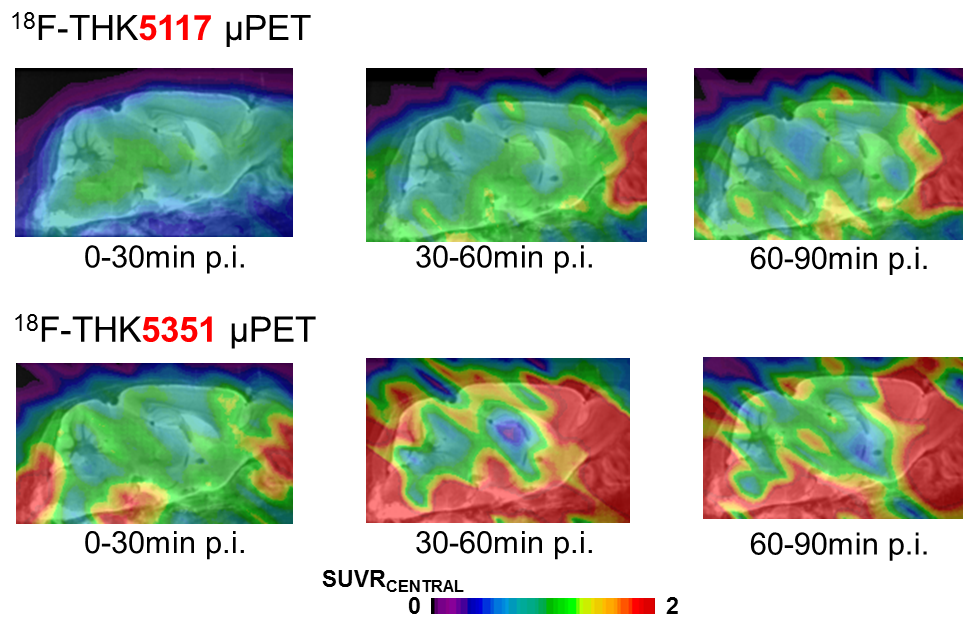


**Suppl. Fig.3:** Head to head comparison of ^18^F-THK5117 and ^18^F-THK5351 PET in a single WT mouse. Images were generated by scaling with a central cerebral reference region (midbrain, thalamus, striatum).
